# Ion Current Rectification and Long-Range Interference in Conical Silicon Micropores

**DOI:** 10.1021/acsami.2c11467

**Published:** 2022-12-09

**Authors:** Mark Aarts, Willem Q. Boon, Blaise Cuénod, Marjolein Dijkstra, René van Roij, Esther Alarcon-Llado

**Affiliations:** † Center for Nanophotonics, AMOLF, Science Park 109, 1098 XGAmsterdam, Netherlands; ‡ Institute for Theoretical Physics, Utrecht University, Princetonplein 5, 3584 CCUtrecht, Netherlands; § Soft Condensed Matter, Debye Institute for Nanomaterials Science, Utrecht University, Princetonplein 1, 3584 CCUtrecht, Netherlands

**Keywords:** conical micropore, ion current rectification, thin membrane, pore array, pore−pore interaction, electro-osmosis, Poisson−Nernst−Planck−Stokes equation

## Abstract

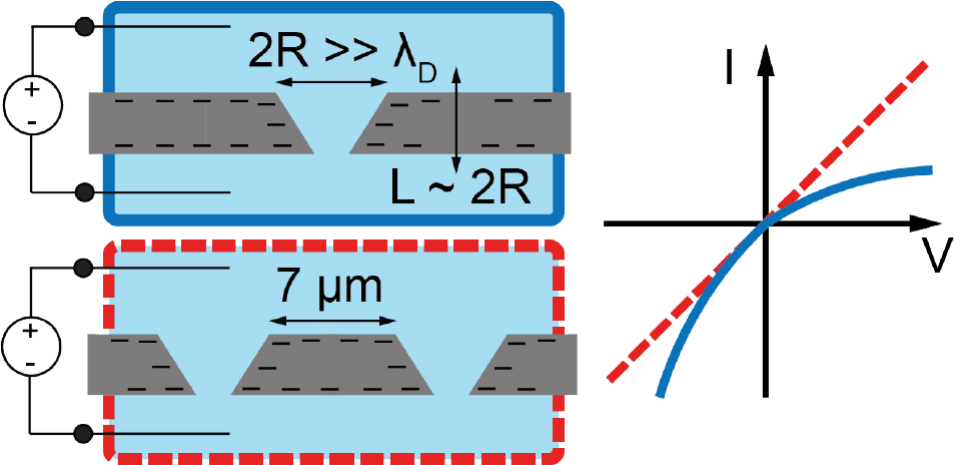

Fluidic devices exhibiting ion current rectification (ICR), or ionic diodes, are of broad interest for applications including desalination, energy harvesting, and sensing, among others. For such applications a large conductance is desirable, which can be achieved by simultaneously using thin membranes and wide pores. In this paper we demonstrate ICR in micrometer sized conical channels in a thin silicon membrane with pore diameters comparable to the membrane thickness but both much larger than the electrolyte screening length. We show that for these pores the entrance resistance is key not only to Ohmic conductance around 0 V but also for understanding ICR, both of which we measure experimentally and capture within a single analytic theoretical framework. The only fit parameter in this theory is the membrane surface potential, for which we find that it is voltage dependent and its value is excessively large compared to the literature. From this we infer that surface charge outside the pore strongly contributes to the observed Ohmic conductance and rectification by a different extent. We experimentally verify this hypothesis in a small array of pores and find that ICR vanishes due to pore–pore interactions mediated through the membrane surface, while Ohmic conductance around 0 V remains unaffected. We find that the pore–pore interaction for ICR is set by a long-ranged decay of the concentration which explains the surprising finding that the ICR vanishes for even a sparsely populated array with a pore–pore spacing as large as 7 μm.

## Introduction

Ionic transport near solid–liquid interfaces can differ drastically from that in bulk due to Coulombic interactions with the surface.^1^ Such interface effects can be used to tailor nanofluidic devices,^2^ finding applications in desalination,^3,4^ ionic circuitry,^5,6^ biochemical sensing,^7−11^ energy harvesting,^12,13^ and neuromorphic signaling.^14−16^ A particularly useful element for directional control of ionic currents is a current rectifier,^9,17−19^ also known as a diode. In fact, the phenomenon of ion current rectification (ICR) has been observed and extensively studied in nanochannels.^20−23^

The ICR originates from an asymmetry in the ionic current along the length of the channel due to a varying relative contribution to the ionic current of the charge-selective electric double layer (EDL) that screens the charge on the channel walls. Typically, ICR is demonstrated in nanoscale conical channels, where EDL overlap occurs on the narrow end of the channel.^21,22^ In general, the ICR mechanism for a geometrically asymmetric, or tapered, channel can be understood by considering that the relative contribution of the salt current through the EDL to the total current is smaller at the wide opening than at the narrow opening.^24,25^ This results in an asymmetry of the transference (i.e., the partial current due to either ionic species). Considering a channel with a negative surface charge on its wall, resulting in an EDL with excess positive ionic charge, an electric field directed toward the narrow end leads to more ions leaving the small opening than entering the large opening (before steady state is reached), resulting in depletion of ions inside the channel and a suppressed conductance.^26^ The opposite is true for an oppositely directed electric field, resulting in accumulation of charge carriers and enhanced conductance. More broadly, the required asymmetry in transference can be introduced not only by geometry but also by a variation of e.g. charge or concentration.^24,26,27^

For application purposes regarding larger scale porous membranes, a low electric resistance of the channel is desirable to mitigate Ohmic losses. Two intuitive ways to construct a channel with low resistance are by making (i) larger openings^26^ or (ii) shorter channels.^28,29^ Considering the accumulation/depletion mechanism described above, recent theoretical work predicts that ICR can also occur in wide channels without overlapping electric double layers, as long as a substantial part of the ionic current is due to surface conductance.^26,30−34^ In fact, ICR in mesoscopic channels^35^ and chemically modified micrometer-sized systems have recently been observed.^32,36,37^ For thin membranes with short channels, on the other hand, it has become clear that the applied potential partially drops outside the channel rather than fully over the channel itself.^29,38−41^ These extended entrance effects give rise to an edge, or access, resistance and become relevant for the behavior of a fluidic pore with a channel length of the order of the diameter, which can either positively contribute to ICR or interfere destructively in arrays of pores.^41−43^ As of now however, such pore–pore interactions are still poorly understood.

In this work we fabricate conical, i.e., geometrically asymmetric, fluidic micropores in thin (2 μm) crystalline silicon membranes, with base and tip radii of *R*_b_ ≈ 1.5 μm and *R*_t_ ≈ 0.5 μm, respectively, such that even the smallest of these channel dimensions is larger than the typical electrolyte screening length by more than an order of magnitude. We demonstrate that these pores exhibit ion current rectification, and we develop an analytical theory for the channel conductance in which the surface potential is the only fit parameter. We stress that the (Ohmic) channel conductance at low applied potentials and ICR are distinct phenomena, and we find that we need a different surface potential to fit the experimental data to these two effects, with both surface potentials being very large, implying a very large surface charge. We interpret the value of these fitted surface potentials as nonphysical and rather attribute this excessively large charge to a contribution of conduction along the planar membrane surface outside the channel at the inlet and outlet of the pore unaccounted for in our model. By connecting this required surface charge to an effective area, we estimate that this membrane surface conduction is relevant up to distances around the pore opening as large as 7.4 μm for Ohmic channel conductance and 15.0 μm for ICR, implying that a larger area around the pore is required for ICR. We test this hypothesis by fabricating a small array of pores with a 10 μm spacing (≈10^6^ pores*/*cm^2^). Indeed, we find that at this low pore density the Ohmic conductance remains unaffected but that the ICR vanishes for the array. Extended entrance effects at the micrometer scale therefore appear to play a significant role in the required asymmetry in ion transport through pores in thin membranes, which we attribute to the long-ranged decay of the electric field outside the pore. This electric field creates a concentration profile with a similar long-ranged, inverse square with distance, decay into the bulk. This scale-free decay introduces long-ranged pore–pore interactions for thin pores, which become particularly relevant in array configurations typical for membranes.

## Experimental Section

**Figure 1:**
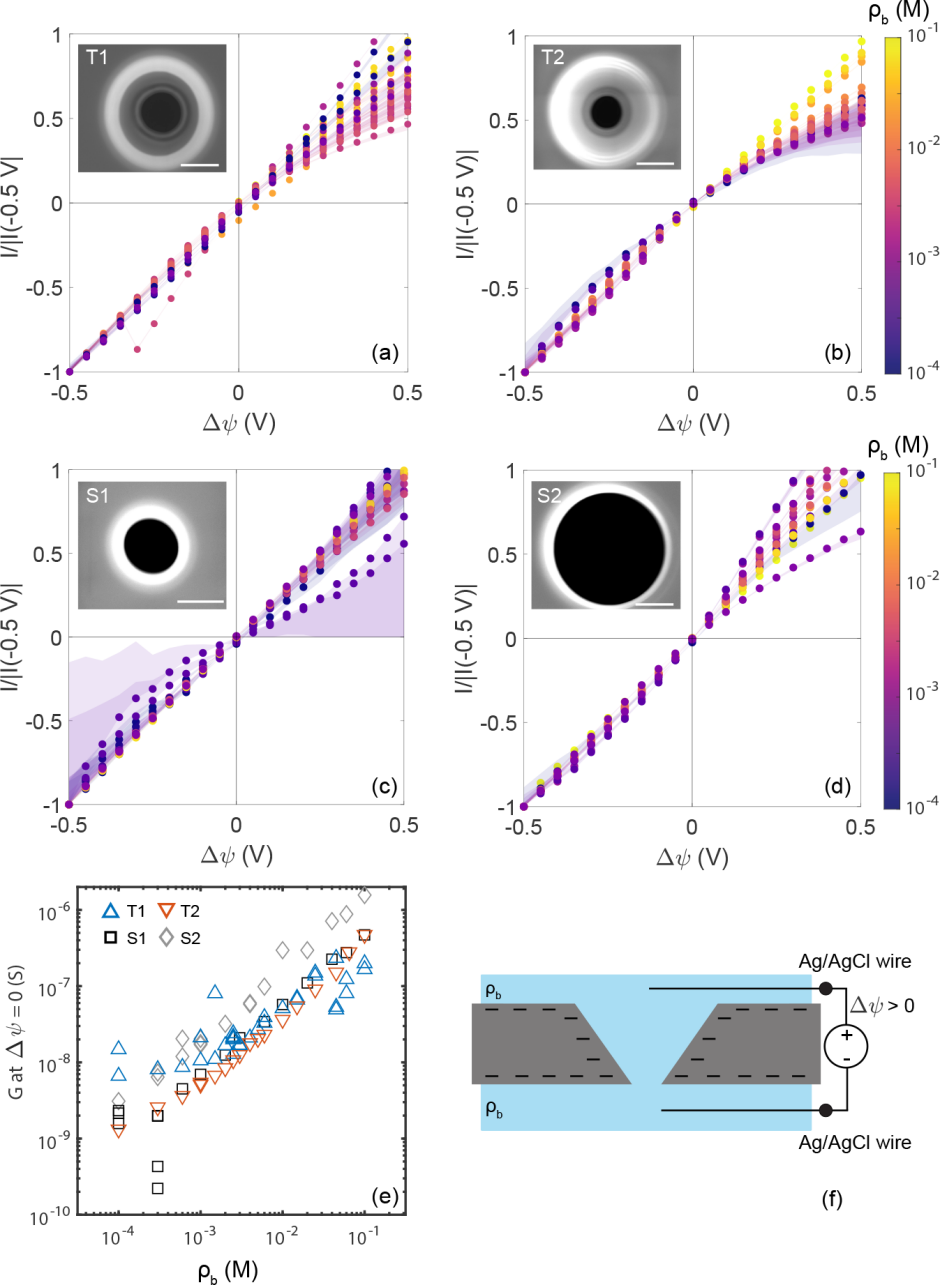
(a–d) Experimental current–voltage (*I*–*V*) curves, normalized at a potential drop of −0.5 V at various KCl concentrations ρ_b_ ∈ {10^–4^–10^–1^} M indicated by the color scale. The shaded region indicates uncertainty in the measurement due to the leakage current obtained from an as-received membrane (SI-3). Systematic current rectification is observed for tapered pores T1 and T2 with the conductivity at +0.5 V being lower than at −0.5 V. The inset shows scanning electron microscopy images of the tapered pores T1 (a) and T2 (b) and straight pores S1 (c) and S2 (d) after fabrication. The scale bars are 1 μm. (e) Conductance of the pores at 0 V as a function of KCl concentration ρ_b_(*M*) . (f) Schematic of the experimental setup where 2 aqueous reservoirs of equal KCl concentration are separated by the membrane with a single pore. The polarity of the potential is such that positive potentials indicate the anode being in the reservoir facing the large opening of the pore.

For our conductance measurements we fabricate single micrometer sized pores, which are either straight or tapered, in 2 ± 0.5 μm thick crystalline silicon membranes using a focused ion beam (FIB). Si is a reliable and cross-compatible platform that allows for precise pore manufacturing. The taper is created by writing concentric circles with decreasing radius, resulting in an asymmetric pore, as verified by atomic force microscopy in SI-1. Conductance measurements are performed by placing the membrane between two aqueous reservoirs containing KCl of equal bulk concentration (ρ_b_) and applying a potential between the reservoirs using Ag/AgCl wire electrodes (Figure 1f and SI-2). Of note is the polarity of the applied potential, where positive potentials indicate the anode being in the reservoir facing the large opening of the pore.

We record quasi-static current–voltage (*I*–*V*) curves between −0.5 and 0.5 V (see the Methods section) at different reservoir KCl bulk concentrations ρ_b_ ranging from 10^–1^ to 10^–4^ M for 4 membranes containing a single pore. The insets of Figure 1a–d show scanning electron microscopy (SEM) images directly after fabrication of the tapered pores T1 (base and tip radii *R*_b_ ≃ 1.5 μm, *R*_t_ ≃ 0.5 μm) and T2 (*R*_b_ ≃ 1.5 μm, *R*_t_ ≃ 0.4 μm) and two straight reference pores S1 (*R*_b_ = *R*_t_ ≃ 0.6 μm) and S2 (*R*_b_ = *R*_t_ ≃ 1.5 μm).

The corresponding *I*–*V* curves are shown as circles in Figure 1a–d, where the colors label the salt concentration ρ_b_. As the magnitude of the current response varies by several orders of magnitude over the salt concentration range, the current is normalized to the value at an applied potential of −0.5 V for visibility. The shaded regions indicate the possible contribution from leakage current through the membrane, averaged from measurements on an as-received membrane without a pore (SI-3). Because of the range in magnitude of the measured currents, this is most relevant for the lowest concentrations and the smallest pore (S1). The conductance at 0 V as a function of concentration is shown in Figure 1e. A linear decrease of the conductance with decreasing concentration is observed with the conductance saturating at ρ_b_ < 1 mM.

At the highest concentrations (yellow, ρ_b_ = 0.1 M), and therefore the smallest Debye length (λ_D_ ≃ 1 nm), all pores show a linear *I*–*V* response, consistent with bulk dominated transport. At lower concentrations, however, conductance through the tapered channels starts to show ion current rectification. It should be noted that even at the lowest concentration, ρ_b_ = 0.1 mM, the electrolyte screening length λ_D_ ≃ 30 nm is much shorter than the smallest tip radius, so that the micropores are well outside the regime of EDL overlap. While some curves for the straight pores S1 and S2 show an erratic deviation from ideal symmetrical conductance, the tapered pores T1 and T2 show systematic rectification, where the conductance at positive potentials is smaller than that at negative potentials.

### Theoretical Framework

In the following, we present a model for the potential-dependent conductance of a tapered pore and obtain a closed-form expression that simultaneously describes Ohmic conductance and ion current rectification. Currently in the literature there are two, complementary, theories for current rectification without EDL overlap for pores with large aspect ratio. The theory by Cengio^30^ and Poggioli^26^ describes ICR through the variation of the surface conductance over the pore length but neglects electro-osmotic flow, while the theory of ref (25) (developed by some of the present authors) does account for this flow but fails at extremely low salt concentrations. Hence both theories are complementary rather than mutually exclusive: refs (26 and 30) are valid at all concentrations, while the theory of ref (25) is valid at all flow rates. We will find that our experiments show characteristic flow-sensitive behavior, and therefore we build on the theory of ref (25). However, to describe our experiments, either theory would need to be extended as the membranes thickness here is similar to the radius of the pores, and the theory therefore has to account for the electric edge resistance which is comparable to the pore resistance. There is a variety of theories available in the literature accounting for Ohmic edge resistance,^2,29,38−40,44^ the most commonly used one by Hall,^39^ which we reproduce here. In this work we will find that edge resistances do not only alter Ohmic conductance but also significantly alter rectification. In the following section we extend the theory of ref (25) to account for edge effects.

**Figure 2:**
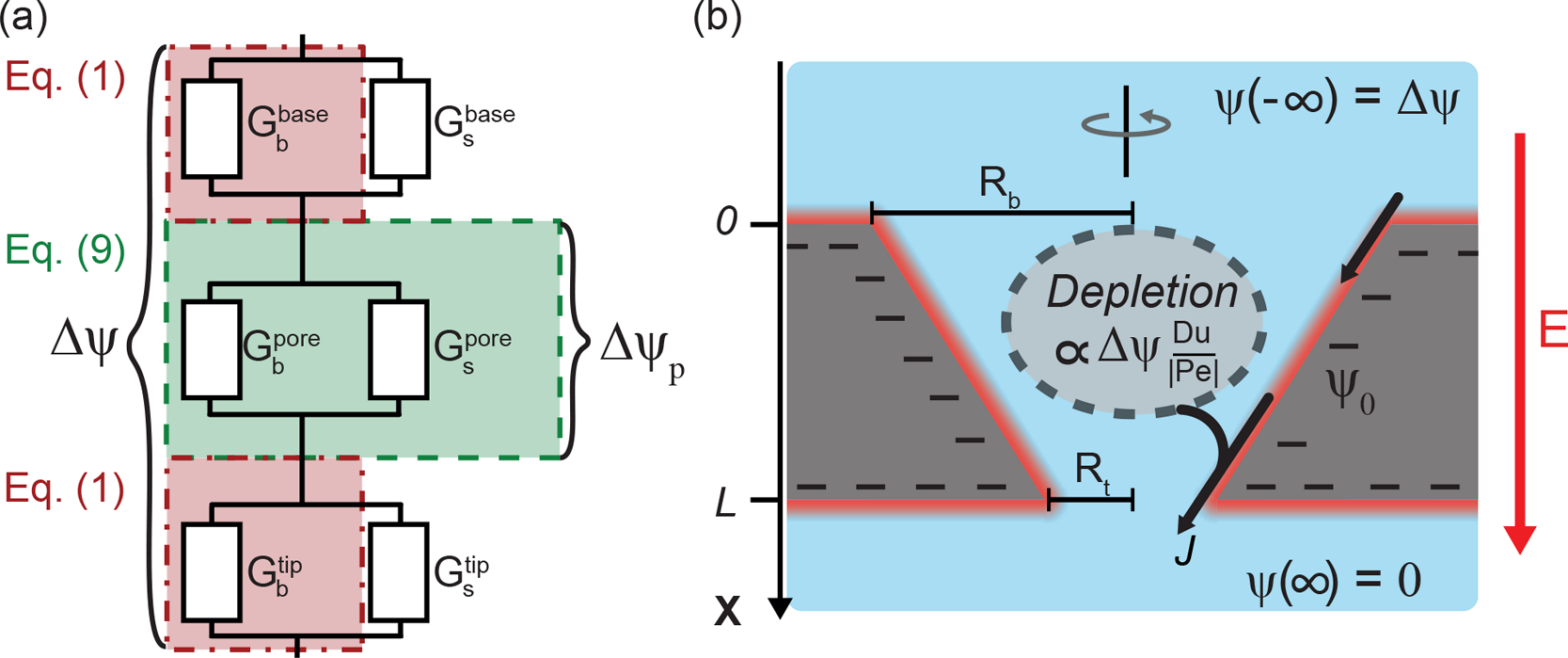
(a) Schematic equivalent circuit of a pore featuring bulk and surface conductance *G*_b_^*i*^ and *G*_s_^*i*^, respectively, at the base, at the tip, and within the pore region. The elements considered in our analytical model are highlighted, where part of the applied potential Δψ drops over the edge resistance (red dash-dotted region), as captured by eq 1. The conductance of the pore with the remaining potential Δψ_p_ (green dashed region) is described by eq 9. (b) Representation of the conical system under consideration with base and tip radii *R*_b_ and *R*_t_, respectively, and an electric field −∂_*x*_ψ = *E* pointed toward the tip. As outlined in the text, the depletion of ions in the channel is proportional to the potential drop Δψ times the ratio of the Dukhin (Du) and Péclet (Pe) numbers as shown in eq 8a and illustrated here for a channel wall with a negative surface charge resulting in depletion for a positive potential drop due to a salt flux *J* (black arrow) through the electric double layer (EDL) that increases toward the tip.

The introduction of non-negligible edge resistances implies an equivalent electric circuit as illustrated in Figure 2a, which considers not only bulk and surface contributions to the conductance inside the pore (*G*_b_^pore^ and *G*_s_^pore^) in parallel^45,46^ but also two base (*G*_b_^base^ and *G*_s_^base^) and tip (*G*_b_^tip^ and *G*_s_^tip^) conductances. Only recently has it been shown that the charged surface outside of the membrane contributes to edge resistance,^2,38^ but unfortunately our model is not able to explicitly account for the base and tip surface conductances (*G*_s_^base^ and *G*_s_^tip^, respectively) even though we will see these charged regions do contribute significantly to both experimental Ohmic conductance and ICR. Instead, we implicitly account for the charge on the outside membrane through an “apparent” (large) surface charge within the pore, inflating the pore surface conductance *G*_s_^pore^ and total conductance *G*. Hence, our large “apparent” surface charge will account for outer-membrane conductance increasing the well-known bulk edge conductances as described by Hall.^39^ These parallel edge surface and bulk conductances are in series with the pore resistances as depicted in Figure 2a, which for the present system parameters ensures that the potential drop over the pore Δψ_p_ is significantly smaller than the total bias Δψ. This decreased potential drop does not only reduce the current through the pore but also lowers the electro-osmotic flow and concentration polarization within the pore. To obtain Δψ_p_ we consider, in cylindrical (*x*,*r*,θ) coordinates, two bulk reservoirs in the half spaces *x* < 0 and *x* > *L* connected by an azimuthally symmetric conical channel of length *L* as depicted in Figure 2b with base radius *R*_b_ at the inlet (*x* = 0) and tip radius *R*_t_ at the outlet (*x* = *L*), such that the radius of the channel reads *R*(*x*) = *R*_b_ – (*x*/*L*)(*R*_b_ – *R*_t_) for *x* ∈ {0, *L*}. The potential drop over the inside of the pore can be calculated using two assumptions: (i) that the electric field at the tip (and base) decays as a monopole −∇ψ ∝ 1/(*r*^2^ + *x*^2^) into the bulk far from the pore (as noted by ref (41)) and (ii) that the electric field within the pore is divergence-free, such that electroneutrality is obeyed. From these assumptions it follows that the pore-potential drop Δψ_p_ = −∫_0_^*L*^∂_*x*_ψ d*x* is given by
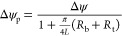
1which we derive and verify in SI-4. We note that the edge resistance is negligible in the long-channel limit *L*/*R*_b_ ≫ 1, as eq 1 reduces to Δψ_p_ ≃ Δψ in this limit. In the geometry of our experiments the reduced potential Δψ_p_ given by eq 1 does not only effectively halve the electric current (as *L* ≈ *R*_b_ + *R*_t_ for our experimental geometries), but as noted it will also significantly influence current rectification. As eq 1 accounts for the influence of bulk-edge resistance (red regions in Figure 2a) for both the electric current and (electro-osmotic) flow and we use an effective surface charge σ as proxy for the surface-edge resistance, from now on our mathematical analysis will pertain only to the inner-pore region (green-shaded region in Figure 2a) therewith following ref (25).

The electric potential difference over the pore Δψ_p_ does drive not only ion fluxes **j**_±_(*x*,*r*) of the cations (*+*) and anions (*−*) but also a fluid flow with a velocity field **u**(*x*,*r*). The salt flux **j**_s_ = **j**_+_ + **j**_–_ and electro-osmotic flow **u** will be key to understanding the electric current **j**_e_ = **j**_+_ – **j**_–_. The resulting salt concentration ρ_s_(*x*,*r*) = ρ_+_(*x*,*r*) + ρ_–_(*x*,*r*) due to the inhomogeneous salt current is of key importance for current rectification, while the space-charge *e*ρ_e_(*x*,*r*) = *e*(ρ_+_(*x*,*r*) – ρ_–_(*x*,*r*)) outside the electric double layer is of secondary importance as was shown in ref (25). The ionic fluxes and concentrations satisfy the Nernst–Planck eqs 2 and 3 which describe diffusion, conduction, and advection, while the electric potential satisfies the Poisson eq 4 in terms of the electric space charge density *e*ρ_e_. The fluid flow in the low-Reynolds number regime of interest here is given by the Stokes eq 5 which includes an electric body force −*e*ρ_e_∇ψ, and the steady-state condition of interest leads to the condition of divergence-free fluxes (eq 6), which all accumulates into
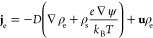
2
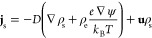
3
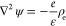
4
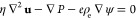
5
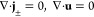
6Here eq 2 shows explicitly that the salt concentration ρ_s_ = ρ_+_ + ρ_–_ determines the electric conductivity of the charge current **j**_e_. To obtain the pore conductance, we consider both reservoirs with a dilute (monovalent) KCl solution of concentration ρ_b_, viscosity η = 1 mPa s, a dielectric permittivity ε = 80ε_0_, with ε_0_ the permittivity of vacuum, and a fixed temperature *T* = 298 K, which is constant throughout the system. Deep into the bulk of the base-connected reservoir, *x* ≪ −*L*, we impose that the K^+^ and Cl^–^ concentrations ρ_±_ = ρ_b_, *P* = *P*_0_, and ψ = Δψ, and deep into the tip-connected reservoir, *x* ≫ 2*L*, we impose ρ_±_ = ρ_b_, *P* = *P*_0_, and ψ = 0. Here the reference pressure *P*_0_ = 1 atm. For K^+^ and Cl^–^ we use equal diffusion coefficients *D* = 1 nm^2^ ns^–1^, which is somewhat smaller than the bulk diffusion constant at 20 °C and 0.1 M.^47,48^ Such a discrepancy between channel and bulk diffusion constants has been noted in ref (40).

In thermodynamic equilibrium with vanishing potential drop between the reservoirs (Δψ = 0) and vanishing fluxes, the PNPS eqs 2–6 reduce to Poisson–Boltzmann theory which describes a diffuse layer of net ionic charge near the surface, known as the electric double layer (EDL) with typical thickness λ_D_ = (8πλ_B_ρ_b_)^−1/2^ and Bjerrum length λ_B_ = *e*^2^/(4πε*k*_B_*T*) ≃ 0.7 nm. Outside this layer all concentrations ρ_±_(*x*,*r*) are equal to ρ_b_ and there is no electric field, – ∇ψ(*x*,*r*) ≃ 0. In equilibrium the surface charge density *e*σ obeys the Gouy–Chapman equation, 2*πλ*_B_λ_D_σ = sinh(*e*ψ_0_/2*k*_B_*T*).^49^ Here ψ_0_ is the surface potential of the channel wall, which we will use as a fit parameter below, taken to be constant between all geometries and at all concentrations thereby implicitly accounting for a concentration-dependent surface charge σ(ρ_b_) due to a salt-concentration-dependent surface reaction.^50,51^

For nonvanishing applied potential drops (Δψ ≠ 0) not only an electric current *I* = 2π*e***x̂**·∫_0_^*R*^**j**_e_*r* d*r* (with **x̂** the unit vector along the *x*-direction) and electro-osmotic flow *Q* = 2π**x̂**·∫_0_^*R*^**u**(*r*)*r* d*r* are driven through the pore but also a salt current *J* = 2π**x̂**·∫_0_^*R*^**j**_s_(*r*)*r* d*r* where the bulk-excess salt current is primarily a conductive current through the EDL. In steady state this salt current must be laterally constant to prevent the buildup of salt through the pore, π*R*^2^(*x*) ∂_*t*_ρ̅_s_ = −∂_*x*_*J* = 0, where ··̅· = 2π(π*R*^2^(*x*))^−1^∫_0_^*R(x)*^···*r* d*r* denotes the cross-sectional average of the salt concentration. The condition of a divergence-free flux (eq 6), which is necessary for a steady-state solution, leads to a differential equation for the cross-sectionally averaged salt concentration for *x* ∈ {0, *L*}
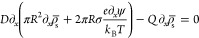
7with the electric field −∂_*x*_ψ = (Δψ_p_/*L*)*R*_b_*R*_t_/*R*^2^(*x*)^25^ and the electro-osmotic flow in a conical channel *Q* = −Δψ_p_(π*R*_t_*R*_b_/*L*)(εψ_0_/η) (as derived beneath eq 2 in ref (25)). In eq 7 the first term represents diffusion of salt through the bulk of the pore, the second term conduction of salt through the EDL, and the third term advection of salt through the bulk of the pore. In a cylinder with constant radius *R* this differential equation reduces to *D*∂_*x*_^2^ρ̅_s_ – *Q*∂_*x*_ρ̅_s_ = 0, which with boundary conditions ρ̅_s_(0) = ρ̅_s_(*L*) = 2ρ_b_ has the trivial solution of ρ̅_s_ = 2ρ_b_. Thus, for straight pores no current rectification is expected. For a conical geometry, the laterally changing radius *R*(*x*) causes lateral variation of conductive currents *D*∂_*x*_(2π*R*(*x*)σ*e*∂_*x*_ψ(*x*)/*k*_B_*T*) ≠ 0, which frustrates the formation of a constant *J* and acts as a nonzero source term. For a negative surface charge, as is typically the case for silica, this source term is negative for Δψ > 0, and thus the salt concentration in the pore decreases. For Δψ < 0 this source term is positive, and thus the salt concentration increases. Solving for the cross-sectional average concentration profile ρ̅_s_(*x*) leads to a nontrivial solution^25^
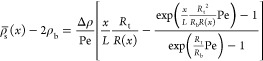
8a
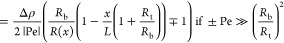
8bwhere the tip Péclet number Pe ≡ −Δψ_p_(*R*_b_/ *R*_t_)(εψ_0_/*D*η) and Δρ ≡ 4(*e*Δψ_p_/*k*_B_*T*)Du(*R*_b_/*R*_t_ – 1)ρ_b_ is a measure for the concentration polarization, with tip Dukhin number Du = σ/(2ρ_b_*R*_t_) . Note that both Pe and Du carry a sign and the diode polarity stems from the sign of the Dukhin number.

Having obtained the salt concentration ρ̅_s_(*x*) in eq 8 the resulting pore conductance *G*(Δψ) = *I*(Δψ)/Δψ is calculated by cross-sectionally integrating eq 2, which results in
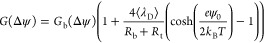
9Here ⟨...⟩ = *L*^–1^∫_0_^*L*^...d*x* denotes the lateral average, and the bulk channel conductance is given by *G*_b_(Δψ) = 4⟨ρ̅_s_⟩*R*_t_*R*_b_(*e*^2^*D*/*k*_B_*T*)/(4*L*/π + *R*_b_+ *R*_t_). This bulk conductance also accounts for the inlet and outlet resistance by incorporation of eq 1 and depends on Δψ through the potential dependence of ⟨ρ̅_s_⟩, which is obtained by numerical integration of eq 8. We note that eq 9 obtained from the PNPS eqs 2–6 has precisely the form expected from the circuit depicted in Figure 2a: it consists of the sum of a bulk and surface (pore) conductance, *G* = *G*_b_ + *G*_s_ (Figure 2a, green), whereas the tip and base conductances (Figure 2a, red) stand in series with the pore and lower the total conductance per eq 1. The surface conductance *G*_s_ = 4*G*_b_⟨λ_D_⟩/(*R*_b_ + *R*_t_)(cosh(*e*ψ_0_)/(2*k*_B_*T*) – 1) will vary with concentration through the dependence of the “channel-weighted” Debye length ⟨λ_D_⟩ ≃ (4πλ_B_⟨ρ̅_s_⟩)^−1/2^. In principle, we could include the advective (streaming current) contribution to eq 9, but its contribution to the surface conductance is proportional to *k*_B_*T*/(4πηλ_B_*D*(cosh(*e*ψ_0_/2*k*_B_*T*) – 1)) ≪ 10^–2^ for all our parameter sets and hence can be neglected.^52^

It is important to note that while the advective contribution to the electric current *I* can be neglected, the advective contribution to the salt current *J* (eq 7) is key to current rectification as for ICR the flow rate determines the characteristic voltage Δψ_c_, known as the knee voltage for diodes. When Δψ_c_ ≫ |Δψ|, the conductance is Ohmic (*G*_0_), while for ±Δψ ≫ Δψ_c_ the limiting diode conductance (*G*_±_) has been reached. From eq 8 it can be seen that for large flow |Pe| ≫ (*R*_b_/*R*_t_)^2^ the concentration profile ρ̅_s_(*x*) – 2ρ_b_ ∝ Δρ/|Pe| is potential-independent, and hence per eq 9 this limiting conductance *G*_±_ has been reached. Hence, the characteristic potential Δψ_c_ is set by the potential for which Pe = (*R*_b_/*R*_t_)^2^, yielding
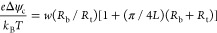
10where *w* = *eD*η/(*k*_B_*T*ε|ψ_0_|) is the (dimensionless) ratio of the ionic to electro-osmotic mobility that quantifies the competition between the rate by which conduction adds ions to the concentration profile ρ̅_s_(*x*) and electro-osmotic flow removes them. Note that this ratio depends only on electrolyte properties and surface potential, and it is not influenced by the geometry whatsoever, being constant (*w* ≃ 0.3) over all our geometries and concentrations. The term in the square brackets of eq 10 accounts for edge resistance and can be set to unity in the long-channel limit. While eq 9 can be used to describe the conductance for arbitrary Δψ by straightforward numerical integration of eq 8a, a more convenient closed form for the limiting conductances *G*_±_ can be found when neglecting the second (surface) term for the electric conductance *G*(Δψ) (eq 9). This approximation therefore neglects surface conductance entirely and subsequently integrating eq 8b yields
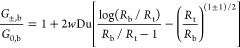
11As such, current rectification is defined by the ratio *G*_–_/*G*_+_ = ICR where, as before, Du = σ/(2ρ_b_*R*_t_) represents the ratio of salt transport in the EDL with respect to salt transport in the bulk. The bracketed term of eq 11 fully captures the effect of geometry on the concentration profile ρ̅_s_(*x*). This last term is zero for *R*_t_ = *R*_b_, positive for *G*_+_, and negative for *G*_–_ and reflects the influence of geometry on diode polarity. Equation 11 also straightforwardly gives a simple and convenient analytic expression for the ICR = *G*_–_/*G*_+_.

In the following sections we consider the small and large potential limits of eq 9 to interpret our experimental data by first considering the measured (Ohmic) conductance *G*_0_ at small potential drops |Δψ| < Δψ_c_ and then to describe ICR, which is given by the ratio of conductances *G*_–_/*G*_+_ in the limit of large positive (+) or negative (−) potential drops for ±Δψ ≫ Δψ_c_.

## Ohmic Conductance

**Figure 3:**
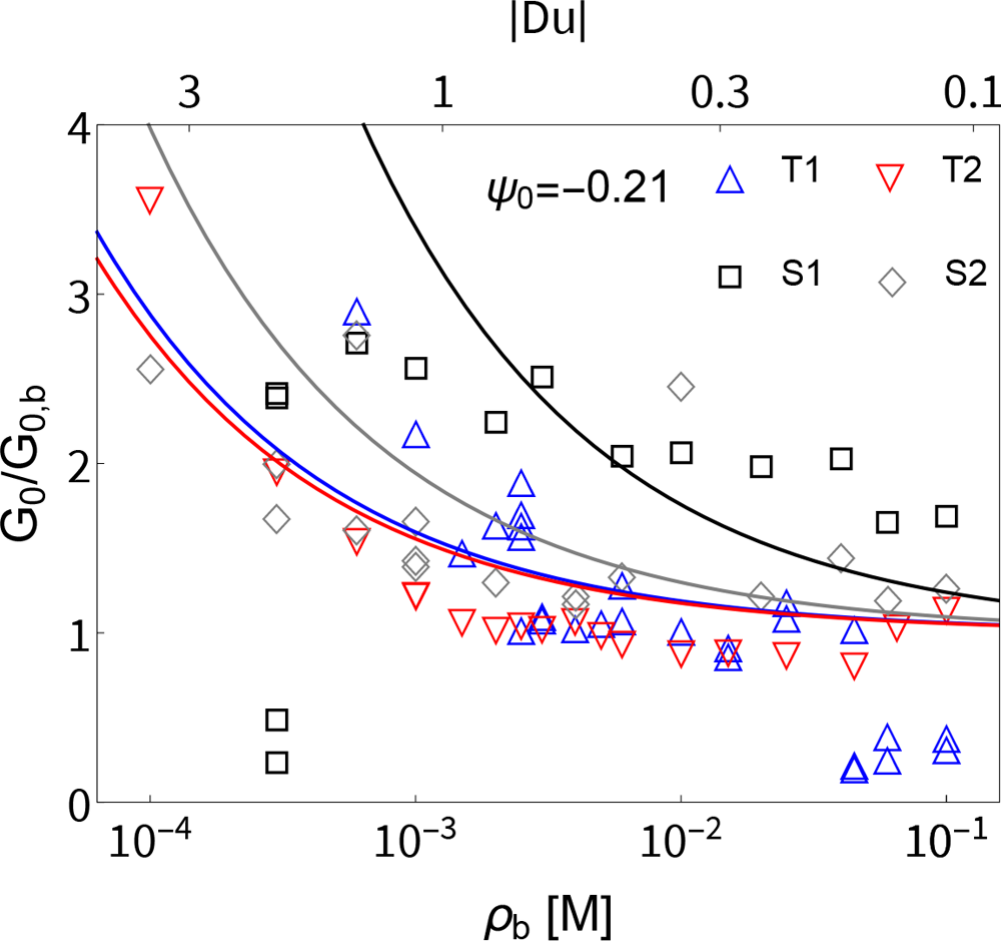
Ohmic conductance *G*_0_ in units of the bulk conductance *G*_0,b_ as a function of the bulk concentration ρ_b_ (lower axis) and Dukhin number Du = σ/(2ρ_b_*R*_t_) (upper axis) with tip radius *R*_t_ = 0.5 μm representative for the tapered pores T1 and T2 and straight pores S1, but not for the straight pore S2 with radius *R*_t_ = 1.5 μm. The symbols denote the experimental measurements, and the lines with corresponding colors are plotted using eq 9 with a large surface potential ψ_0_ = −0.21 V (see text). As expected, the conductance converges to the bulk conductance at high concentration while it increases to triple the bulk conductance at low concentrations due to the contribution of surface conductance.

First we consider the Ohmic conductance, *G*_0_, which is found at small potential drops |Δψ| < Δψ_c_. In this case the laterally averaged concentration equals the bulk concentration ⟨ρ̅_s_⟩ = 2ρ_b_. Hence, *G*_0_ is given by eq 9 where the bulk Ohmic conductance *G*_0,b_ = 8*R*_t_*R*_b_(*e*^2^*D*/*k*_B_*T*)/(4*L*/π + *R*_b_ + *R*_t_)ρ_b_, and the surface Ohmic conductance *G*_0,s_ is determined by the equilibrium Debye length λ_D_ = (8πλ_B_ρ_b_)^−1/2^. In this regime we find that eq 9 reduces to several well-known results depending on the geometry. The conductance of a long conical channel with negligible surface conductance is retrieved when *L* ≫ *R*_b_ > *R*_t_ ≫ λ_D_,^25^ the Hall conductance of a thin cylindrical pore with negligible surface conductance is retrieved when *L* ≃ *R*_b_ = *R*_t_ ≫ λ_D_,^39^ and the conductance of a long cylindrical channel^52^ is obtained when *L* ≫ *R*_b_ = *R*_t_ > λ_D_. Hence, eq 9 extends these classical results to short pores with unequal tip and base radii. Figure 3 shows the experimental Ohmic conductance obtained as *G*_0_ = (*I*(0.05 V) – *I*(−0.05 V))/0.1 V together with our theoretical model eq 9 for all four channels T1, T2, S1, and S2, where we use ψ_0_ = −0.21 V as it provides the closest match to the data for all concentrations and geometries. Note that the classical long-channel theory that neglects entrance resistance through eq 1 would overestimate the conductance by a factor of about 2 for our parameters, as the effective potential drop is nearly halved (0.46 < Δψ_p_/Δψ < 0.68) in our experimental geometries. This reduction of the effective potential drop highlights the importance of edge resistances.

It should be emphasized that the experimental conductance in Figure 3 is normalized by the theoretical bulk conductance *G*_0,b_ from eq 9, which is determined by both pore geometry and electrolyte properties. At high concentrations this representation highlights that the surface conductance is negligible, as the conductance in units of *G*_0,b_ approaches unity and the experimental data for different geometries collapse into a single curve. Note that eq 9 properly accounts for the inlet and outlet resistance at the highest concentration. Some of the deviation between experimental data and theory is attributed to morphological changes due to clogging over the course of the experiments (see, for instance, the SEM image of T1 after the experiments in SI-5).

At low concentrations, ρ_b_ < 1 mM, both the theoretical and experimental conductance rapidly increase as the concentration decreases, which is due to the surface conductance *G*_0,s_ contribution increasing with the increasing Debye screening length. We observe that the concentration at which surface conductance becomes comparable to bulk conductance occurs when the tip Dukhin number approaches unity, *G*_0,s_/*G*_0,b_ ∝ Du = σ/(2ρ_b_*R*_t_) ≃ 1, which for both T1 and T2 occurs near ρ_b_ ≃ 2 mM for ψ_0_ = −0.21 V. The experimental variation of conductance with concentration roughly scales as the inverse square of the concentration, increasing by a factor of 3 when the concentration is decreased by a factor of 10. This scaling can only be understood by using a concentration-independent ψ_0_ as opposed to a concentration-independent surface charge density σ. With constant σ the Dukhin number scales as Du ∝ ρ_b_^–1^ and introduces a surface conductance which varies by orders of magnitude in our concentration range, which is not observed. Instead, when using a constant ψ_0_, the surface charge scales as σ ∝ 1/λ_D_ according to the Gouy–Chapman equation, in which case the proper scaling Du ∝ ρ_b_^–1/2^ is immediately obtained. The existence of a constant surface potential implies that a chemical reaction is responsible for the surface charge varying with salt concentration. However, while Figure 3 indeed shows that the experimental conductance qualitatively follows the inverse square-root scaling, there is a minor quantitative deviation. We attribute this chiefly to charge regulation beyond the constant-potential model of the silica–water interface,^53^ which could introduce variations in ψ_0_ by a factor of ∼3 in the range 10^–1^–10^–4^ M for silica.^50,51,54^ We have not included this concentration effect as there is no unanimous quantitative measurement of charge regulation for silica^50,51,53−55^ and as to prevent overfitting.

## Ion Current Rectification

**Figure 4:**
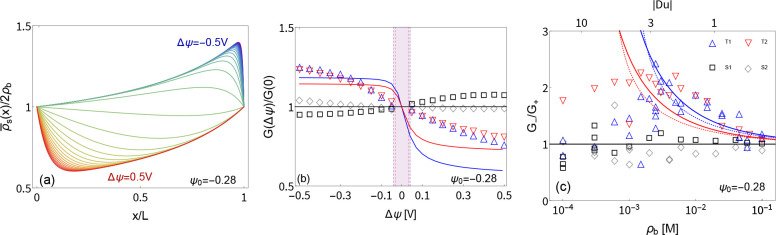
(a) Concentration profiles ρ̅_s_(*x*)/2ρ_b_ for geometry T1 (see text) at a bulk concentration ρ_b_ = 6 mM as calculated by eq 8 for Δψ between −0.5 and 0.5 V with a step size of 0.03 V. Depletion occurs for positive potentials (red) while concentration increases at negative potentials (blue). (b) Conductance normalized by the conductance at Δψ = 0 at varying potentials and the same concentration as in (a) where the different symbols represent the tapered (T1, T2) and straight (S1, S2) channels, and lines are plotted using the combination of eq 9 and eq 8a both with a surface potential of ψ_0_ = −0.28 V. Vertical lines demarcating the shaded area are placed at the characteristic voltage Δψ_c_ ≃ ±0.05 V with color corresponding to the respective geometry. Most variation of the experimental conductance occurs within the shaded region, after which the conductance converges to the limiting conductances *G*_±_. (c) Current rectification given by the ratio *G*_–_/*G*_+_ which for experiments is taken as *G*(±0.5 V) with varying concentration (lower axis) and Dukhin number for *R*_t_ = 0.5 μm (upper axis). Solid lines are plotted with our approximation eq 11 which neglects surface conductance, and dotted lines are from the full solution using the combination of eq 9 and eq 8a both using ψ_0_ = −0.28 V. Peak experimental current rectification is reached near Du ≃ 3 while solid lines grow monotonically with Du.

We now turn to large potential drops where |Δψ| ≫ Δψ_c_ (eq 10) where we observe significant current rectification for tapered pores T1 and T2 and the conductance has converged to its limiting value *G*_±_ (eq 11). Current rectification in conical pores is well established to be due to the salt concentration in the pore changing with the applied potential^25,56−58^ as described in the Theoretical section. The dependence of the laterally averaged salt concentration ⟨ρ̅_s_⟩ on Δψ as in eq 8 in conjunction with our expression for the conductance (eq 9) immediately results in a voltage-dependent conductance. In Figure 4a we use eq 8 to plot the salt concentration profiles ρ̅_s_(*x*)/(2ρ_b_) in the conical pore T1 for Δψ between −0.5 and 0.5 V and a concentration ρ_b_ = 6 mM. It can be seen that for negative voltages there is a buildup of ions, while for positive voltage drops the pore becomes depleted. This increase and decrease, characteristic of the conical geometry, is highly dependent on the applied voltage but converges to a limiting concentration profile for which inhomogeneous conduction is balanced by advection. These limiting concentration profiles can deviate up to 50% from the bulk concentration and in turn significantly modulate the voltage-dependent conductance as can be seen in Figure 4b. Here we compare *G*(Δψ) from eq 9 with the experimental conductance normalized by the experimental Ohmic conductance *G*(Δψ = 0) . We observe two plateaus of high and low conductance for the theoretical curves at large negative and positive voltages for the tapered pores. The transition between the two regimes occurs at the borders of the shaded region |Δψ| ≤ Δψ_c_ ≃ 0.05 V, beyond which the conductance quickly converges to the limiting conductance *G*_±_. In SI-7 we show more plots at different concentrations for comparison, which all show the same typical S-shaped curve with the exception for curves at ρ_b_ < 1 mM for which the experimental variation is larger due to leakage currents as discussed in the Experimental Section.

In Figure 4c we plot the experimental ICR against concentration and tip Dukhin number, together with results based on both eq 11 (solid) and the combination of eq 9 with eq 8a (dashed) using a fitted surface potential ψ_0_ = −0.28 V in both cases. Theoretical and experimental ICR obey the same inverse square root scaling *G*_–_/*G*_+_ ∝ ρ_b_^–1/2^ as was also observed for Ohmic conductance, which is again due to the concentration dependence of Du at fixed surface potential ψ_0_. Interestingly, we find that eq 11 is a good approximation for the combination of eq 9 with eq 8a. The unexpected quality of our approximation eq 11 is a result of a cancellation of errors: an increase of Ohmic conduction due to surface conductance decreases ICR while the variation of surface conductance with concentration polarization increases ICR.

At low concentrations the inverse square root dependence of ICR breaks down around ρ_b_ ≃ 2 mM, where the experimental ICR peaks while our theory predicts that ICR should keep increasing with decreasing concentration. Such a peak in ICR has been previously observed in long microchannels^29,33,56^ and assigned to the emergence of EDL overlapping at low concentrations. Here the concentration depletion in the pore only allows for minor EDL overlap at the tip, as the screening length is at least an order of magnitude smaller than the tip size in our experiments. Other theoretical works predict a peak in ICR at 1 < Du < 10,^26,30^ which is attributed to salt transport in the EDL dominating the total salt transport, an effect that is not captured in our model. In SI-9 we plot the pore selectivity as defined in ref (30) for our tapered geometries with ψ_0_ = −0.21 V and find a maximum near ρ_b_ = 2 mM, in line with our experiments.

## Discussion of the Large Surface Potential

Both conductance *G*_0_ and current rectification *G*_–_/*G*_+_ are visually fitted using ψ_0_ as the only fit parameter which we keep constant for all geometries and concentrations, yielding ψ_0_ = −0.21 V for *G*_0_ and ψ_0_ = −0.28 V for *G*_–_/*G*_+_. However, the surface chemistry of the silica interface is well studied, and a much lower surface potential between −0.03 V and −0.1 V is expected in our experimental conditions.^55^ While surface potentials may vary quite significantly between different measurement methods, protocols and even subsequent measurements,^55^ a discrepancy that exceeds 0.1 V is excessive. From the Gouy–Chapman equation we find that a pore with a surface potential between −0.21 and −0.28 V would contain approximately 15–60 times more charge than for a typical literature surface potential of −0.07 V. Such a large discrepancy cannot be explained by subtle experimental factors. Induced charge due to membrane capacitance may partly explain the large apparent charge at large applied potentials (Δψ).^59^ However, previous work found that induced charge on 55 nm thick silicon membranes is comparable to the surface charge of silica.^60^ As the membrane capacitance and therefore the induced charge scales with the inverse of membrane thickness, we expect the (material specific) influence of induced charge to be minor in the 2 μm thick membranes used here. Furthermore, the large apparent charge required to explain Ohmic conductance at Δψ ∼ 0, can not be justified by induced charge. We therefore assume that this deviation stems from our theoretical model not including all of the key physics.

In our analysis we exclude the charge on the planar membrane outside the pore, effectively neglecting an edge EDL conductance *G*_s_^base^ and *G*_s_^tip^ as depicted in Figure 2a. Other authors have noted that this region on the outside of the pore can contribute to both the Ohmic conductance^38,40^ and the current rectification^41,42^ for thin pores with *R*_b_/*L* ≤ 1. The charge on the outside of the membrane not only increases the edge conductance as noted by^2,38^ but surprisingly can also induce excess ICR.^41^ This excess ICR is due to a radial electric field driving an inhomogeneous salt current through the EDL ouside of the pore, leading to accumulation/depletion of salt in the reservoir as we demonstrate in SI-6. While excess ICR and excess conductance both occur in the EDL outside of the pore, they are distinct phenomena whose scaling and characteristic length scales may differ qualitatively, explaining why our experimental *G*_0_ and *G*_+_/*G*_–_ are better accounted for with two different surface potentials, ψ_0_ = −0.21 V and ψ_0_ = −0.28 V, respectively.

Considering that the charge on the outside of the membrane can contribute to both ICR and increased conductance, we suggest to assign the excess charge from the large, fitted, surface potential ψ_0_ to the charge located on the planar membrane outside of the pore. We estimate this charged-surface area *A*_out_ outside the pore as *A*_out_ = *A*_pore_((σ_app_/σ_lit_) – 1), where *A*_out_ is the (circular) area outside of the pore contributing to entrance-surface conductance, *A*_pore_ is the surface area of the conical pore, σ_app_ = sinh(ψ_0_*e*/2*k*_B_*T*)/(2πλ_B_λ_D_) is the apparent (Gouy–Chapman) surface charge density resulting from the fitted surface potential (with ψ_0_ = −0.21 V for Ohmic conductance and ψ_0_ = −0.28 V for ICR), and σ_lit_ = sinh(−0.07*V*(e/2*k*_B_*T*))/(2πλ_B_λ_D_) is the surface charge density as calculated from a literature surface potential of −0.07 V. With these values we find that the outer-membrane EDL within a radius of about 15.0 μm from the pore center contributes to ICR, while a shorter radius of only 7.4 μm contributes to Ohmic conductance. This latter value closely corresponds to the Dukhin length σ/(2ρ_b_) ≃ 7 μm at 10^–4^ M, which was predicted by refs (2 and 38) to set the length scale for (Ohmic) outer-membrane surface conductance.

**Figure 5:**
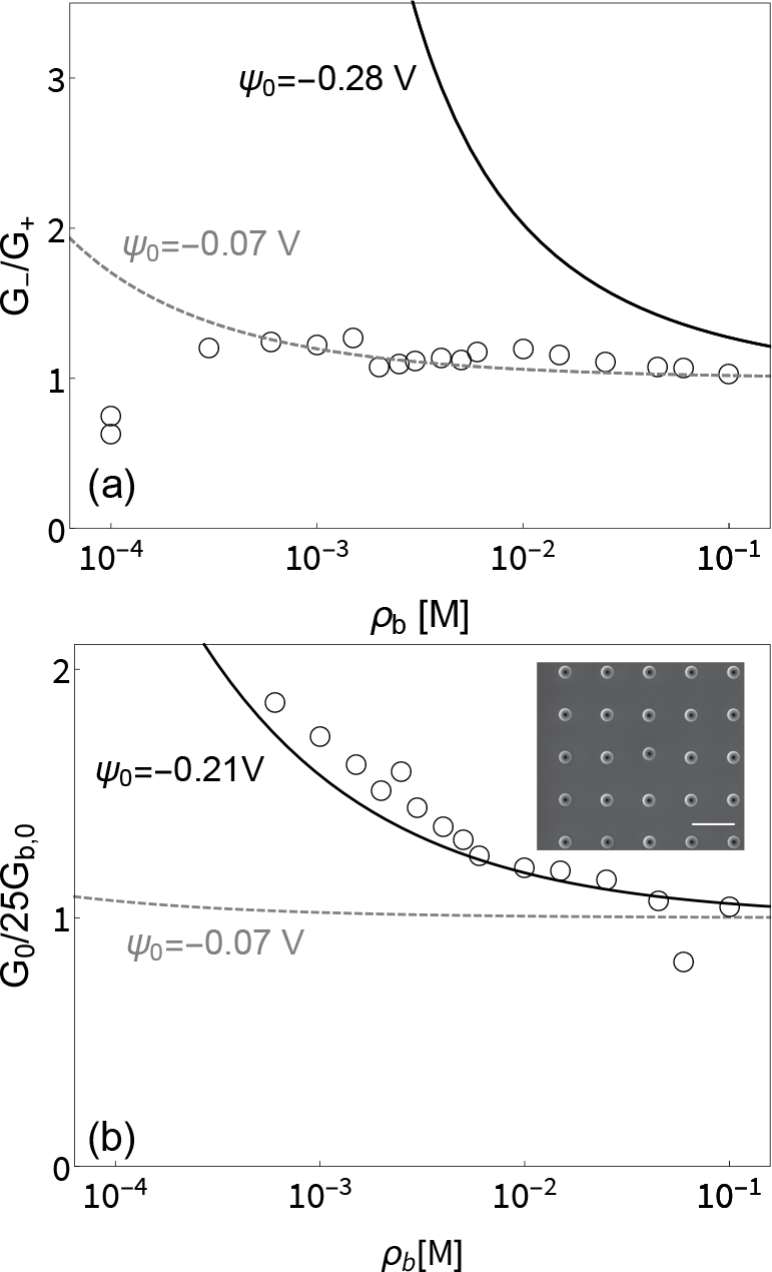
(a) Current rectification *G*_–_/*G*_+_ as a function of concentration (lower axis) for an array of 25 pores. The symbols denote the experimental measurements, and the lines are plotted using eq 11 using a surface potential of ψ_0_ = −0.28 V (black, continuous) and ψ_0_ = −0.07 V (gray, dashed). No current rectification is found in the experiment, in line with a pore with a surface potential of ψ_0_ = −0.07 V. (b) Dimensionless Ohmic conductance *G*_0_ in units of the bulk conductance *G*_0,b_ in the same representation as in Figure 3 with lines plotted using eq 9. The measured conductance corresponds essentially to 25 times the conductance of a single pore with ψ_0_ = −0.21 V. The inset of (b) shows an SEM image of the array directly after fabrication; the scale bar is 10 μm.

To verify this larger surface contribution of 15 μm for ICR, we derive a solution for the concentration polarization far from the pore in SI-6 and find that the concentration profile in the bulk obeys a long-ranged inverse square law decay ρ_s_(*x*,*r*) ∝ (*r*^2^ + *x*^2^)^−1^ like the electric field with a prefactor proportional to the inverse aspect ratio *R*_b_/*L*. This prefactor indicates that outer-membrane concentration polarization only occurs for short pores, while the inverse square decay indicates that the concentration profile decays over lengths much larger than the radius. Both these observations support the hypothesis that surface charge far from the pore can contribute to current rectification for low-aspect ratio pores. Unfortunately, we were unable to construct a theory simultaneously accounting for concentration polarization inside and outside the pore, while numerical (COMSOL) calculations of the full PNPS (eqs 2–6) proved unstable. To experimentally test our hypothesis, we construct a 5 × 5 array of conical pores with dimensions *R*_t_ ≃ 0.35 μm, *R*_b_ ≃ 1.4 μm, and *L* ≃ 2 μm with a spacing of 10 μm between the pore centers (≈10^6^ pores/cm^2^). In Figure 5a we show the ICR calculated with eq 11 using the literature surface potential ψ_0_ = −0.07 V (dashed line) and the large surface potential obtained from the single pore fitted with ψ_0_ = −0.28 V (solid line) together with the experimental data for the array (symbols). We observe that ICR is greatly reduced for the array, virtually disappearing over the whole concentration range. In contrast, the Ohmic conductance of the array is essentially 25 times the single pore conductance given by eq 9 as shown in Figure 5b. Considering that the single pore results are based on the fitted value of ψ_0_ = −0.21 V, the surface conductance therefore remains excessively large compared to the expectation at a literature surface potential of ψ_0_ = −0.07 V (solid line). In line with our hypothesis, these observations therefore surprisingly imply that the charge on the outside of the membrane contributes over a smaller range to conductance than to ICR, so that interference only occurs for the latter at this spacing.

## Conclusion

To summarize, we have presented experimental and theoretical results on ion current rectification (ICR) in tapered micropores connecting aqueous KCl solutions, leading to three main results.

(i) We demonstrate the existence of ICR in conical micropores fabricated in crystalline silicon membranes without further chemical modification at KCl concentrations where the (bulk) electrolyte screening length is much smaller than the pore size and which is absent in straight cylindrical pores.

(ii) We derive an expression for the conductance of short conical pores accounting for both the EDL within the channel as well as the edge resistance at the tip and base of the pore. These edge resistances approximately halve the Ohmic conductance in our experimental geometries. Our expression (eq 9) reverts to the Hall conductance in the case of thin cylindric pores,^39^ conical conductance in the case of long cones,^25^ and the well-known conductance of straight cylinders with EDL’s for large aspect ratio channels.^52^ We find an expression for the characteristic voltage at which current rectification occurs, Δψ_c_ ≃ 0.05 V in our geometries, and find a new closed-form expression (eq 11) for the limiting ICR at large potential drops. While, like other authors, we find that rectification scales with the Dukhin number, our expression contains two new dimensionless terms: the ratio *w* of the ionic and electro-osmotic (fluid) mobility and a term describing the influence on geometry which solely depends on the tip-to-base ratio. Using two different surface potentials (ψ_0_ = −0.21 V for Ohmic conductance and ψ_0_ = −0.28 V), our theory closely matches our experimental results for all but the lowest concentrations and largest potential drops. In this regime of extreme depletion minor EDL overlap occurs at the tip, invalidating the starting assumption of nonoverlapping EDL’s in our theory.

(iii) Finally, we discuss the physical interpretation of the surface potential ψ_0_ which is our sole fit parameter. Our fitted surface potential is excessively large compared to literature values and should not be interpreted as the actual potential but rather as an apparent surface potential. This apparent potential is inflated by the contribution of charge on the outside of the membrane, a region explicitly excluded from our theoretical description. We estimate from the fitted ψ_0_ that charge on the membrane surface within about 7.4 μm of the pore contributes to (Ohmic) conductance at low potentials and within 15.0 μm to ICR at larger potentials. We test this hypothesis by fabricating an array of 25 pores with a 10 μm separation of the pore centers (≈10^6^ pores/cm^2^) with no overlap of the low potential (Ohmic) interaction length and large overlap of the high potential (ICR) interaction length. While we observe no pore–pore interference for Ohmic conduction at low potentials, we indeed find that ICR vanishes in the array, in agreement with our hypothesis. The interaction length for Ohmic conduction is known to be set by the Dukhin length^2,38^ while for ICR we show that a long-ranged, inverse-square-distance decay determines the pore–pore interaction, in line with our experimental observations. For thin membranes this apparent contribution of the charge on the outside of the membrane to both surface conductance and ICR may be beneficial for single pores; however, these contributions could be detrimental in densely packed arrays that would be desirable for applications. Further investigation of the interaction length for outer-membrane conductance and ICR with different pore densities is therefore particularly relevant. Here, the presented crystalline silicon membranes provide an attractive platform compatible with conventional fabrication methods for e.g. creating homogeneous pore walls through wet etching or engineering of the pore behavior by modification of the outer-membrane surface.

## Methods

### Pore Fabrication

Crystalline silicon membranes were purchased from Norcada (SM5200N, thickness 2 ± 0.5 μm). Pores were milled in the membrane with a focused ion beam (FEI Helios Nanolab 600, Ga ions), with tapered pores created by milling concentric circles. Scanning electron miscroscopy images were made in the same system.

### Current Measurements

*I*–*V* measurements were conducted in a homemade cell consisting of two reservoirs, with the membrane fixed in between (as shown in SI-2). The reservoirs are filled with aqueous solutions with equal concentration of KCl (99.99% from Sigma-Aldrich, in Milli-Q ultrapure water). Measurements are done using a Ag/AgCl wire electrode in each reservoir, connected to a potentiostat (CH760E) in a two-electrode configuration with the working electrode facing the large opening (base) for the tapered channels. Quasi-static *I*–*V* curves were recorded using staircase voltammetry from −1 to 1 V and back, with potential steps of 0.05 V and with the system set to rest for 150 s at −1 V before starting the measurement. Each potential is maintained for a period of 10 s, with the current recorded for the last 0.5 s. Data shown in Figure 1 are obtained from averaging 1–4 cycles.

### Electrode Preparation

Ag/AgCl wires were fabricated following the protocol in ref (61) by immersing Ag wires (0.35 mm diameter, 99.99%) in 0.1 M nitric acid to remove the native oxide and then rinsed with ultrapure water. The cleaned wire was using as a working electrode in a three-electrode setup with a platinum counter and quasi-reference electrode in an aqueous solution of 3 M KCl. The Ag wire was coated with AgCl by applying 2 V vs the Pt wire QRE for 10 min.
