# Supplementary material for: Ion Current Rectification and Long-Range Interference in Conical Silicon Micropores
Source: ACS Appl Mater Interfaces. 2022 Dec 9;14(50):56226–36. doi: 10.1021/acsami.2c11467 (PMC9782324; doi:10.1021/acsami.2c11467)
Supplement: Supplementary file 1 — am2c11467_si_001.pdf [file am2c11467_si_001.pdf]

Supporting Information: Ion Current Rectification and Long-Range Interference in Conical Silicon Micropores

Mark Aarts,[†,§] Willem Q. Boon,[‡,§] Blaise Cuénod,[†] Marjolein Dijkstra,[¶] René van Roij,[‡] and Esther Alarcon-Llado[*,†]

†*Center for Nanophotonics, AMOLF, Science Park 109, Amsterdam, Netherlands*

‡*Institute for Theoretical Physics, Utrecht University , Princetonplein 5, 3584 CC Utrecht*

¶*Soft Condensed Matter, Debye Institute for Nanomaterials Science, Utrecht University, Princetonplein 1, 3584 CC Utrecht, The Netherlands*

§*Authors contributed equally*

E-mail: e.alarconllado@amolf.nl

Supporting Information 1: Atomic Force Microscopy profile of tapered feature made with Focused Ion Beam

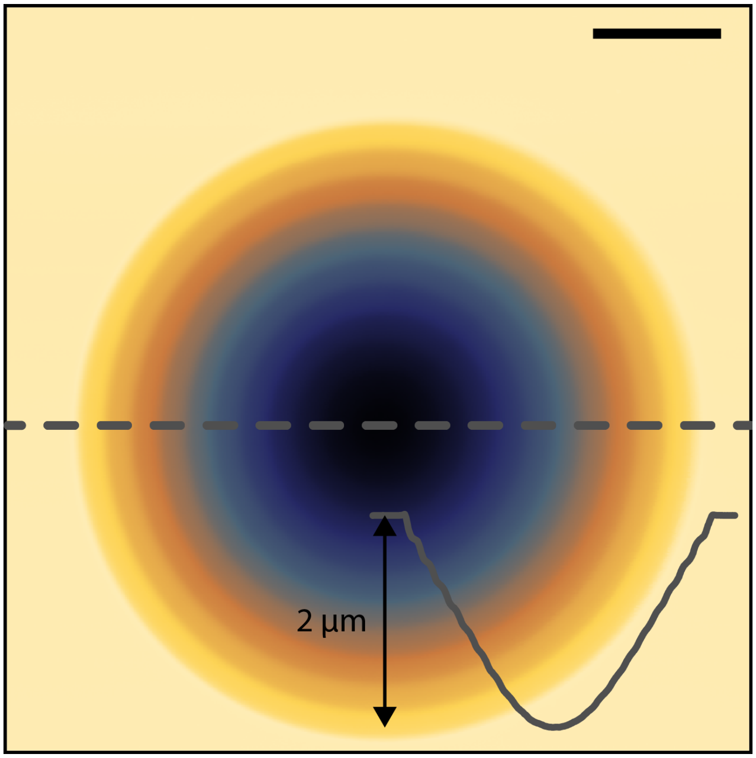

Figure S1: AFM image of a tapered hole with 5 μm radius made with FIB at the edge of the membrane on top of the supporting frame. The centerline profile (grey dashed line) is depicted in the image (grey solid line), with the total depth of the hole being 2 μm. The colour scale runs from light yellow (high) to dark blue (low), the scale bar is 2 μm

Atomic Force Microscopy (AFM) image of a profile resulting from our Focused Ion Beam milling (FIB) protocol writing concentric circles, shown for a hole with a 5 μm base radius. Milling is done at the edge of the membrane where it is supported by a silicon frame, allowing AFM imaging. The image and inset profile illustrate the smooth profile. The depth of the hole is 2 μm. This depth appears to be set by the underlying frame as the milling rate was found to be much slower once the frame is reached.

Supporting Information 2: Illustration of the experimental setup

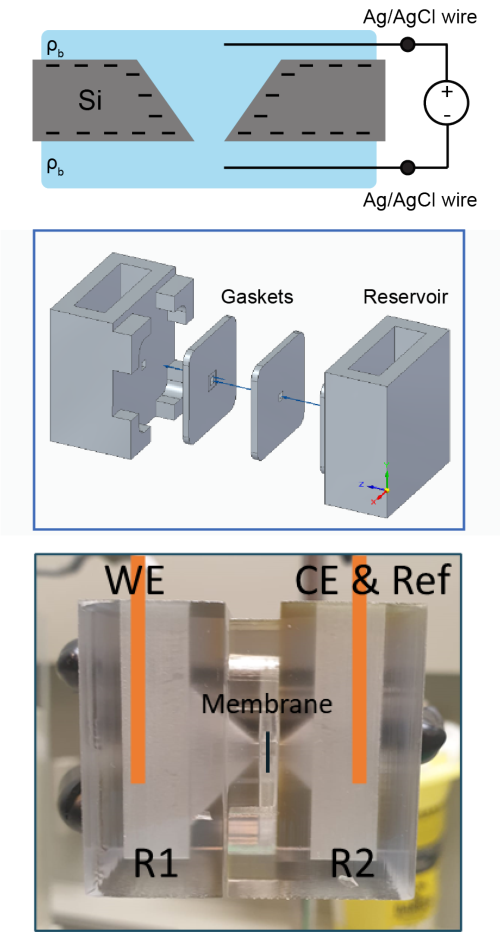

Figure S2: **(top)** Schematic of the experimental setup, with 2 reservoirs of equal concentration ρ_b of KCl on either side of the membrane. **(middle)** 3D drawing of the setup, consisting of 2 electrolyte reservoirs and 2 PDMS gaskets. **(bottom)** Photo of the experimental setup with the membrane and electrode positions indicated. The working electrode (WE) is connected to a Ag/AgCl wire placed in reservoir R1 facing the large opening of the pore, and the counter and reference electrodes (CE and ref) are both connected to the second Ag/AgCl wire in reservoir R2, facing the small pore opening. All components are clamped together using a laboratory clamp.

Schematics and photo of the experimental setup. The setup consists of 2 electrolyte reservoirs (3D printed in a transparent commercial polymer *VeroClear*) and 2 Polydimethylsiloxane (PDMS) gaskets. The reservoirs have a tapered opening leading towards the membrane, to facilitate filling of the channel without trapping air. The PDMS gaskets (thickness $\approx$ 1 mm) were cast in an aluminium mould to obtain the hole in the center, and a recess to fit the membrane. Measurements

are done in a 2-electrode configuration with the working electrode (WE) connected to a Ag/AgCl wire in the reservoir facing the large pore opening (R1) and the counter,- and reference electrode (CE and Ref) connected to a Ag/AgCl wire in the reservoir facing the small pore opening (R2), as indicated in the photo. The membrane is placed between the gaskets and the setup is tightly squeezed and held together by a laboratory clamp.

Supporting Information 3: Current-voltage characterization of as-received membrane

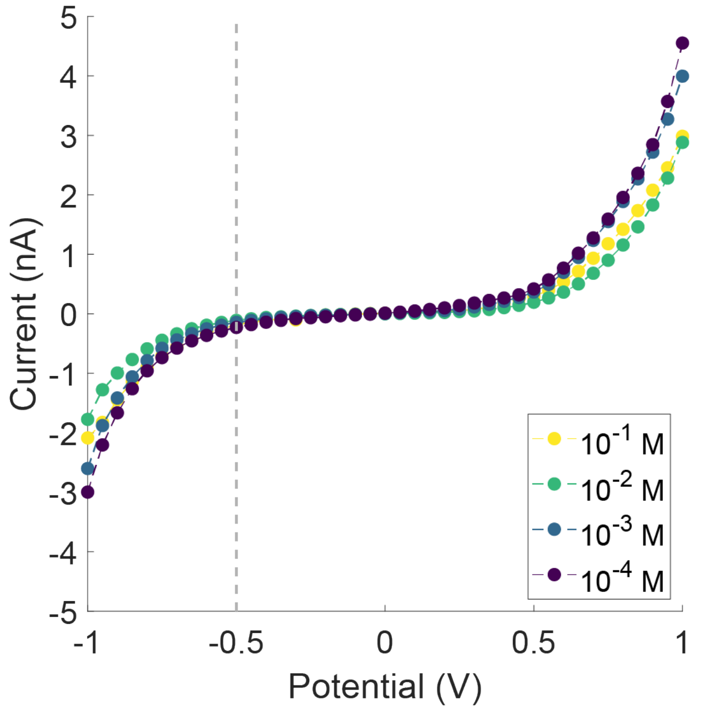

Figure S3: I-V reference curves determining the leakage current of an as-received membrane, without any pore. The leakage current is the same for different concentrations of KCl, as indicated by the legend. The grey dashed line at -0.5 V indicates the potential at which data in Fig. 1 in the main text is normalized.

Current-voltage (I-V) characterization of an as-received membrane for different concentrations of KCl. However, the leakage current becomes significant at potentials $|\Delta\psi| \geq 0.5$ V, in particular considering our measurements at low concentrations where the total current through the pore is in the 1-10 nA range. The leakage current does not show a clear concentration dependence, despite differing slightly between different measurements. It is worth mentioning that the setup is disassembled for filling with a different concentration, illustrating the robustness of the sealing by the gaskets for subsequent measurements at the potentials considered in the main text. The average of these measurements is used as lower bound of the shaded region in Figure 1(a-d) in the main text to illustrate the potential contribution by the leakage current, capped at $I = 0$ for 2 of the curves in Figure 1(c).

Supporting Information 4: Calculation of the electric electric field

In this section we will calculate the electric field $-\nabla\psi$ not only within the pore but also in both reservoirs. This analysis supposes that the space charge ρ_e outside the EDL is negligible, ensuring that the electric field is divergence free $\nabla^2\psi = 0$. We assume (i) that the electric field in the bulk reservoirs far from the pore $\sqrt{r^2+x^2} \gg R_b$ is isotropic and decays like an electric monopole by the inverse square law $\propto 1/(r^2+x^2)$ and, (ii) that no electric field permeates the channel walls. The far-from-pore assumption (i) solution breaks down in the near-pore region $r^2+x^2 \simeq R_b^2$ where the electric field diverges and therefore a characteristic cut-off length scale for this asymptotic decay has to be identified. Natural length scales would be the tip and base radii R_t and R_b near which the far-from-pore solution fails, but to obtain quantitative agreement with numerical calculations we have to multiply the base and tip radii by $\pi/4$. Choosing this factor will also reproduce the exact resistance for a cylindrical, 2D-pore as derived by Hall.[S1] Following assumption (ii) the field inside the conical pore $0 < x < L$ scales as $\partial_x\psi \propto 1/(\pi R^2(x))$ as the total, radially integrated, lateral electric field has to be constant. Combining these expression we find the electric field over the centre axis $r = 0$ is given by

$$-\partial_x\psi(x,r=0) = \begin{cases} \frac{\alpha\pi^2}{4}\Delta\psi\left(\frac{\pi}{4}R_b - x\right)^{-2} & \text{if } x<0; \\ \alpha\Delta\psi R^{-2}(x) & \text{if } 0<x<L; \\ \frac{\alpha\pi^2}{4}\Delta\psi\left(x+\frac{\pi}{4}R_t - L\right)^{-2} & \text{if } x>L, \end{cases} \tag{S1}$$

where the constant length $\alpha = R_bR_t/(4L+\pi(R_b+R_t))$ can be found by requiring that the electric field is continuous at the pore edges and the total potential drop equals $\psi(-\infty)-\psi(\infty) = \Delta\psi$ and the maximum electric field (at the tip) is equal to $\alpha\Delta\psi/R_t^2$. We have chosen to evaluate the electric field on the center line where the field is purely axial as to give an explicit expression for one of the vector components of $-\nabla\psi$. Furthermore we note that this component is of greatest interest as it is responsible for the axial currents through the pore. In Fig.S4 we compare the analytic expression of the electric field (Eq.(S1)) over the central axes and find good agreement with numerical results.

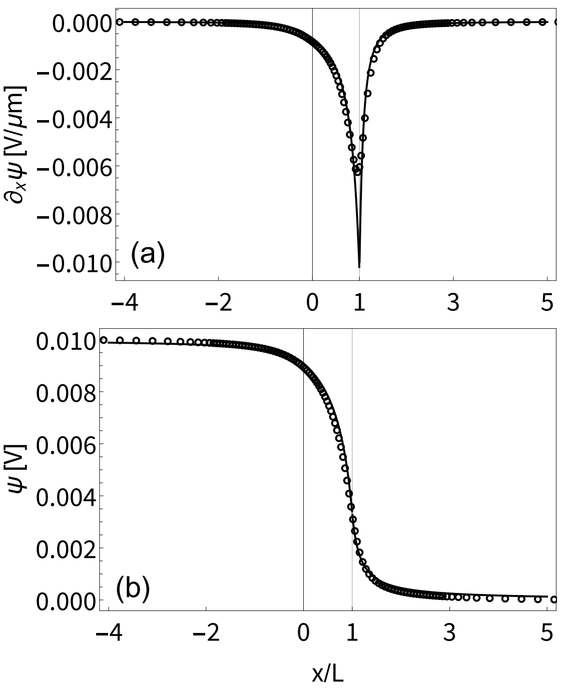

Figure S4: (a) Gradient of the electric potential $\partial_x \psi(x)$ and (b) electric potential $\psi(x)$ along the central axis $r = 0$ for the T1 geometry (see the main text) at a vanishing surface potential and $\rho_\mathrm{b} = 1$ mM for $\Delta\psi = 0.01$ V with symbols from numerical calculations and lines resulting from Eq.(S1). This parameter set is representative for our experimental system at high concentrations, where surface conductance is negligible. The base and tip locations are at $x = 0$ and $x = L$ denoted by vertical lines. There is good agreement between numerical and analytic results.

Supporting Information 5: Scanning Electron Microscope image of pore T1, after measurements

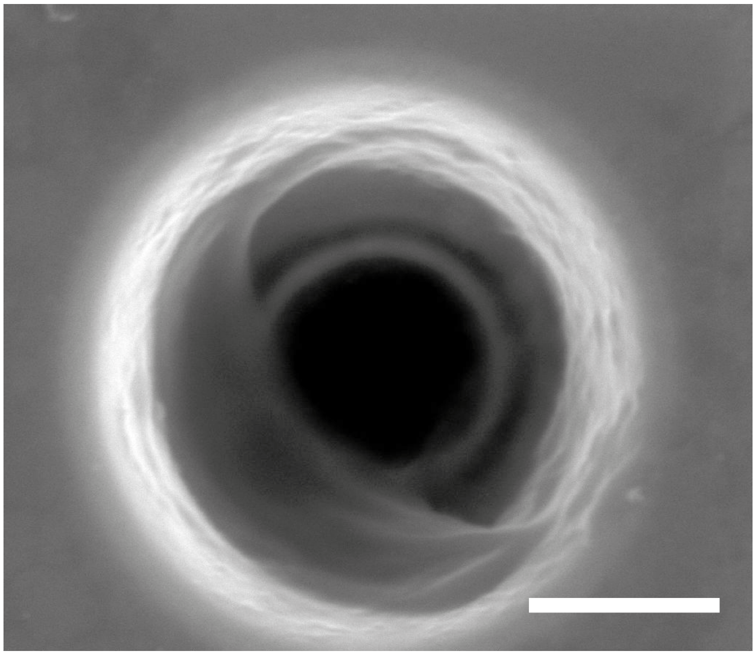

Figure S5: SEM image of the tapered pore T1 after the measurements. The scale bar is 1 μm

Fig.S5 shows a scanning electron microscope (SEM) image of the tapered pore T1 in the main text, after the measurements. Both partial clogging and roughening of the pore are observed thereby changing the pore geometry. Hence clogging will change the pore conductance, which likely explains the variation of conductance over a series of experiments. The chronology of experiments is from low to high-concentration.

Supporting Information 6: Inlet-outlet concentration polarization

In this section we will construct a far-from-pore solution demonstrating concentration-polarization in the bulk reservoir with $x < 0$ connected to the pore base. Instead of the cylindrical (x, r, θ) coordinates used in the main text it will be convenient to treat the problem in spherical (s, ϕ, θ) coordinates with $s^2 = x^2 + r^2$ and $\cos\phi = x/\sqrt{x^2 + r^2}$. We consider fluxes far from the pore opening $s \gg L$ where the electric field Eq.(S1) simplifies to $\partial_s \psi \simeq -\alpha\pi^2\Delta\psi/(4s^2)$. The aim here is to calculate $\hat{\rho}_s(s)$, with $\hat{\cdots} = (2\pi s^2)^{-1}\int_0^{2\pi}\int_{\pi/2}^{\pi} \cdots s^2 \sin(\phi)\mathrm{d}\phi\mathrm{d}\theta$ the average over a hemisphere centered on the origin extending in the bulk with radius s. The hemispherical average $\hat{\rho}_\mathrm{s}(s)$ is not representative for the local concentration $\rho_\mathrm{s}(s, \theta, \phi)$ which is expected to have a large ϕ dependence as the electric double layer is localized at $\phi = \pi/2$ and the far-from-pore Landau-Squire[S2] solution for the fluid flow $\mathbf{u}(s, \phi)$ is much larger at $\phi = \pi$ than at $\phi = \pi/2$. Both these complicating factors will expectedly yield a concentration profile with larger deviations from bulk concentration near the membrane surface $\phi = \pi/2$ compared to $\phi = \pi$. Nevertheless our expression for $\hat{\rho}_\mathrm{s}(s)$ can explain two experimental observations, (i) concentration-polarization in the bulk reservoir is expected in the small-pore limit $L/R_\mathrm{b} \simeq 1$ and (ii) the concentration profile extends long distances into the bulk, exhibiting long-ranged, inverse-square decay.

Integrating the radial component $j_{\mathrm{s},s}(s, \phi)$ of the salt flux to obtain the total salt flux $\hat{J}(s) = \int_0^{2\pi}\mathrm{d}\theta\int_{\pi/2}^{\pi}\mathrm{d}\phi \sin(\phi)s^2 j_{\mathrm{s},s}(s, \phi)$ and imposing the stationarity condition $\partial_s\hat{J} = 0$ we find a differential equation for the concentration $\hat{\rho}_\mathrm{s}(s)$ averaged over a hemisphere,

$$D(2\pi\partial_s(s^2\partial_s\hat{\rho}) - \frac{\pi^3\alpha\sigma}{2s^2}\frac{e\Delta\psi}{k_\mathrm{B}T}) + Q\partial_s\hat{\rho}_\mathrm{s} = 0, \tag{S2}$$

with s denoting the radius of the hemisphere over which the concentration is averaged, α being defined below Eq.(S1) and where $\int_0^{2\pi}\mathrm{d}\theta\int_{\pi/2}^{\pi}\mathrm{d}\phi\sin(\phi)s^2\rho_\mathrm{e}(s, \phi) = -2\pi s\sigma$ stems from hemispherical charge-neutrality. Furthermore we made the approximation that flow can be considered to be isotropic which combined with incompressibility yields $2\pi s^2 u_s(s) = -Q$, where the minus

sign was added so that a radially inward flow in the bulk reservoir results in a positive Q following the convention in the main text. Solving Eq.(S2) for $\hat{\rho}_{\rm s}$ with bulk boundary conditions $\hat{\rho}_{\rm s}(\infty) = \partial_s \hat{\rho}_{\rm s}(\infty) = 0$ we find

$$\hat{\rho}_{\rm s}(s) - 2\rho_{\rm b} = \Delta\rho_{\rm res}\left(\exp\left(\frac{l_{\rm Pe}}{s}\right) - \frac{l_{\rm Pe}}{s} - 1\right) \overset{s\gg|l_{\rm Pe}|}{\simeq} \frac{\Delta\rho_{\rm res}}{2}\left(\frac{l_{\rm Pe}}{s}\right)^2, \tag{S3}$$

where the measure for the concentration profile extending into the reservoir is

$$\Delta\rho_{\rm res} = \frac{\pi\sigma}{4 l_{\rm Pe}^2}\frac{e\Delta\psi}{k_{\rm B}T}\left[\frac{R_{\rm b}R_{\rm t}}{4L/\pi + R_{\rm b} + R_{\rm t}}\right], \tag{S4}$$

with the Péclet length $l_{\rm Pe} = Q/2\pi D$ signifying the distance from the origin at which advective and diffusive transport rates are equal. Note that Q and hence the Péclet length has a sign. The term in square brackets vanishes in the long-channel-limit as the electric-field in the bulk and correspondingly $\Delta\rho_{\rm res}$ go to zero in this limit, which shows that no pore-pore interactions are expected for long, thin pores. While our solution was specifically derived for the base reservoir with $x < 0$ and $\phi \in [\pi/2, \pi]$, our solution Eq.(S3) is valid in the tip-connected reservoir with $x > L$ and $\phi \in [0, \pi/2]$ when interchanging $-\Delta\rho_{\rm res}$ for $\Delta\rho_{\rm res}$ and $-l_{\rm Pe}$ for $l_{\rm Pe}$ as the flow and electric field are anti-symmetric between tip- and base-connected reservoirs. Due to the anti-symmetry of the far-from-pore solutions the depletion in one reservoir leads to a compensating excess in the other reservoir for $s \gg |l_{\rm Pe}|$ (where the asymptotic decay is independent from $l_{\rm Pe}$) and the only contribution to ICR is expected from the near-pore region. The unphysical divergence of the concentration profile near the pore for positive (inward) flows $s \ll l_{\rm Pe}$ prevents us from connecting the far-from-pore solution to the near-pore region. In this regime the large inward flow sweeps up the concentration profile and concentrates it in the near-pore region where the far-from-pore solution breaks down.

As the flow is always inwards for one of the two reservoirs there is no scenario where the far-from-pore solution can be used to describe the entire experimental system. We note that this

focusing of the concentration profile near the base for positive flows also complicates numerical calculations: a significant effort was made to obtain numerical calculations from COMSOL, however no finite-element system could be created that was stable beyond a very narrow parameter regime. Our numerical calculations always showed reservoir concentration polarization in some form. The near-pore solution is expected to very sensitively depend on all experimental length scales, including Péclet and Dukhin length. A "holistic" model describing the entire concentration profile extending over both the reservoirs and pore would be desirable as it would allow for quantitative predictions without an "apparent" ψ_0 as fit parameter. This problem is left for future study.

Supporting Information 7: Conductances at four different concentrations

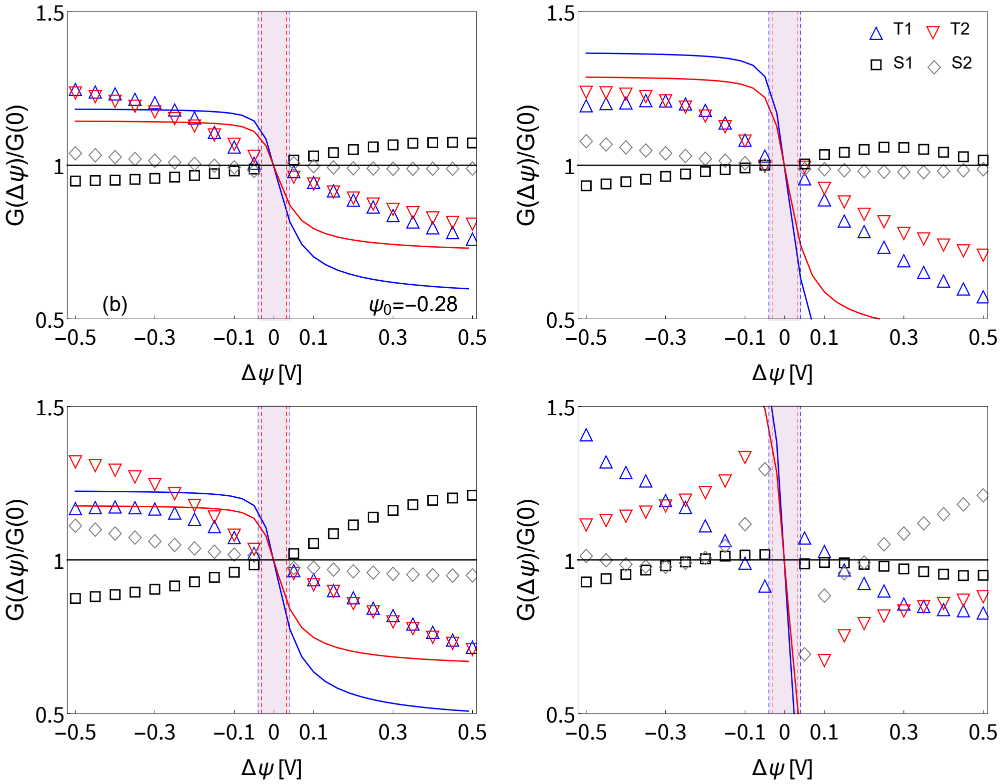

Figure S6: Reproduction of Fig.4(a) in the main text at different concentrations with (a) $\rho_b = 1.5$ mM, (b) $\rho_b = 10$ mM, (c) $\rho_b = 6$ mM and (d) $\rho_b = 0.3$ mM. Figure (a) represents the best agreement between theory and experiment we could obtain, (b) and (c) are typical for our experimental results while (d) shows the large experimental variation typical at low concentrations.

Supporting Information 8: More fits of experimental data

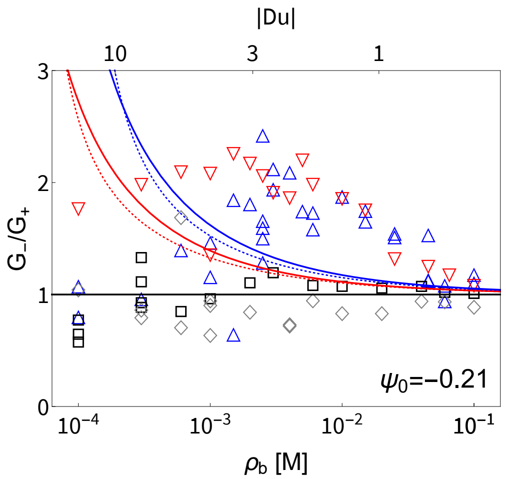

Figure S7: Current rectification G_-/G_+ with the surface potential $\psi_0 = -0.21$ V obtained from the fit on Ohmic conductance instead of the ideal fitted surface potential for ICR $\psi_0 = -0.28$ V. The quality of the fit has decreased significantly compared to Fig. 4 in the main text, but the same qualitative trend can be observed.

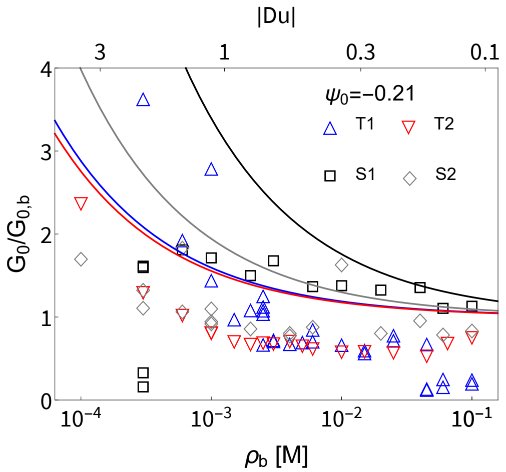

Figure S8: Fit of Ohmic conductivity G_0 in units of the bulk conductance $G_{0,\mathrm{b}}$ with a diffusion constant $D = 1.5\ \mathrm{nm}^{-2}\ \mathrm{ns}^{-1}$ instead of $D = 1\mathrm{nm}^{-2}\ \mathrm{ns}^{-1}$ as presented in Fig.3 of the main text.

Supporting Information 9: Selectivity from literature

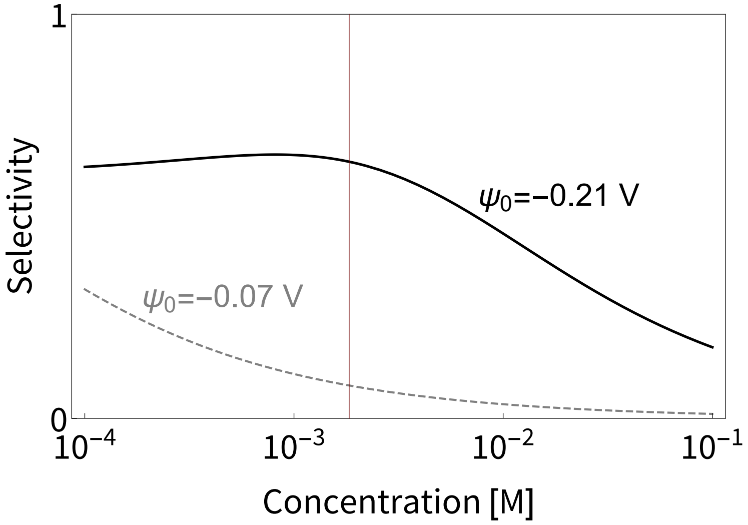

Figure S9: Selectivity as defined by Ref.[S3] for our experimental geometry T1 with a surface potential $\psi_0 = -0.21$ V (solid) and $\psi_0 = -0.07$ V (dashed). The selectivity shows a maximum at $\rho_\mathrm{b} \simeq 2$ mM (vertical line) for $\psi_0 = -0.21$ V in line with our own experimental and theoretical results. Using a literature surface potential of $\psi_0 = -0.07$ V little selectivity is expected. This shows that other theories also require an excessively high surface potential to explain the observed ICR.

References

(S1) Hall, J. E. Access Resistance of a Small Circular Pore. *The Journal of general physiology* **1975**, *66*, 531–532.

(S2) Landau, L. D.; Lifshitz, E. M. *Fluid Mechanics: Landau and Lifshitz: Course of Theoretical Physics, Volume 6*; Elsevier, 2013; Vol. 6.

(S3) Dal Cengio, S.; Pagonabarraga, I. Confinement-Controlled Rectification in a Geometric Nanofluidic diode. *The Journal of Chemical Physics* **2019**, *151*, 044707.
